# Host BAG3 Is Degraded by Pseudorabies Virus pUL56 C-Terminal ^181^L-^185^L and Plays a Negative Regulation Role during Viral Lytic Infection

**DOI:** 10.3390/ijms21093148

**Published:** 2020-04-29

**Authors:** Chuang Lyu, Wei-Dong Li, Shu-Wen Wang, Jin-Mei Peng, Yong-Bo Yang, Zhi-Jun Tian, Xue-Hui Cai

**Affiliations:** State Key Laboratory of Veterinary Biotechnology, Harbin Veterinary Research Institute of Chinese Academy of Agricultural Sciences, Haping Road No.678, Harbin 150069, China; htylv123@126.com (C.L.); lwd12378@163.com (W.-D.L.); Wangshuwen157@126.com (S.-W.W.); pjm7614@163.com (J.-M.P.); yangyongbo@caas.cn (Y.-B.Y.); tzj@hvri.ac.cn (Z.-J.T.)

**Keywords:** *UL56* gene, alpha-herpesvirus, WW-domain, PPxY motif, leucine

## Abstract

Bcl2-associated athanogene (BAG) 3, which is a chaperone-mediated selective autophagy protein, plays a pivotal role in modulating the life cycle of a wide variety of viruses. Both positive and negative modulations of viruses by BAG3 were reported. However, the effects of BAG3 on pseudorabies virus (PRV) remain unknown. To investigate whether BAG3 could modulate the PRV life cycle during a lytic infection, we first identified PRV protein UL56 (pUL56) as a novel BAG3 interactor by co-immunoprecipitation and co-localization analyses. The overexpression of pUL56 induced a significant degradation of BAG3 at protein level via the lysosome pathway. The C-terminal mutations of ^181^L/A, ^185^L/A, or ^181^L/A-^185^L/A in pUL56 resulted in a deficiency in pUL56-induced BAG3 degradation. In addition, the pUL56 C-terminal mutants that lost Golgi retention abrogated pUL56-induced BAG3 degradation, which indicates a Golgi retention-dependent manner. Strikingly, BAG3 was not observed to be degraded in either wild-type or UL56-deleted PRV infected cells as compared to mock infected ones, whereas the additional two adjacent BAG3 cleaved products were found in the infected cells in a species-specific manner. Overexpression of BAG3 significantly suppressed PRV proliferation, while knockdown of BAG3 resulted in increased viral yields in HEK293T cells. Thus, these data indicated a negative regulation role of BAG3 during PRV lytic infection. Collectively, our findings revealed a novel molecular mechanism on host protein degradation induced by PRV pUL56. Moreover, we identified BAG3 as a host restricted protein during PRV lytic infection in cells.

## 1. Introduction

Pseudorabies virus (PRV), which is one of the most important swine pathogens, belongs to the *Alphaherpesvirinae* subfamily and contains a large (approximately 150 kb) double-stranded DNA genome. The virion is composed of a complex structure including a linear genome surrounded by nucleocapsid, proteinaceous tegument, and a lipid bilayer envelope from the inner side to the outer side, in addition to some host proteins (e.g., Rab GTPases) [1,2]. During the lytic infection of alpha-herpesviruses, the interplay between virus and host proteins contributes to a diversity of influences on the viral life cycle (e.g., viral entry, replication, envelope, and egress) [2,3].

Host BCL-2-associated athanogene (BAG) 3 is a co-chaperone protein of the BAG protein family, and was first isolated and identified as a Bcl-2 binding protein [4,5]. The family members share a highly conserved BAG domain at the C-terminus [5,6]. Among these family proteins, BAG3 is the only one that contains an N-terminal WW-domain [5,6,7]. To date, two BAG3 variants (^81^IIe and ^81^Met) have been discovered in mice. The novel variant (^81^Met BAG3) was reported recently, and the level of genetic variation was sufficient to confer susceptibility to necrosis in mice [8].

The interaction between BAG3 and PPxY motif containing viral proteins is a well-defined event that occurs during a viral infection [7,9,10]. This interaction is involved in modulation of the viral life cycle, and such effects have been observed in alpha-herpesviruses, filoviruses, arenaviruses, and coronaviruses [7,9,10,11,12]. A depletion of BAG3 results in an inhibition of varicella-zoster virus (VZV) replication [9]. In addition, BAG3 is required for the temporal regulation and accumulation of immediate early gene products for augmenting the replication of herpes simplex virus (HSV) 1 through an interaction between ICP0 and BAG3 [10]. These findings suggest that BAG3 can facilitate the replication of alpha-herpesviruses by affecting the immediate early gene products. In contrast, the interaction between BAG3 and PPxY motif containing the matrix protein VP40 in Ebola virus and Marburg virus or Z protein in Lassa fever virus suppresses the budding of VP40 virus-like particles. Thus, BAG3 counteracts the egress and spread of viral particles, which suggests a negative regulatory role [7,11]. Moreover, the inhibition of the *BAG3* gene by RNA interference (RNAi) can lead to a significant suppression of SARS-CoV replication [12]. Thus, host BAG3 can be involved in regulating the viral life cycle via different approaches, which results in either a positive or a negative effect on viral proliferation during multiple processes of lytic infection.

PRV type II membrane protein UL56 (pUL56) has four PPxY motifs [13], which represents a potential BAG3 interactor. Recently, PRV pUL56 has been identified as a virulence-associated factor that contributes to viral dissemination in the rodent nervous system [14]. The HSV1 pUL56 is involved in the maintenance of the viral virulent phenotype through its C-terminal hydrophobic domain [15]. In HSV2, pUL56 interacts with a series of WW-domain containing ubiquitin ligase neuronal precursor cell-expressed developmentally down-regulated (Nedd) 4 family members (e.g., Nedd4 and Itch) [16,17,18]. Moreover, alpha-herpes viral pUL56 homologs can induce the degradation of several host proteins via the PPxY motif in both transfected and infected cells [18,19,20,21].

In the current study, we first identified BAG3 to interact with PRV pUL56 through the WW-domain−PPxY interaction module. BAG3 was found to be degraded by pUL56 in transfection experiments. The precise molecular mechanism underlying pUL56-induced BAG3 degradation was further studied. In contrast to the previous investigation showing that the PPxY motif was responsible for HSV or PRV pUL56-induced host protein degradation, our data indicated that the C-terminal ^181^L-^185^L contributed to PRV pUL56-induced BAG3 degradation. Moreover, this process was on dependence of a specific Golgi retention of pUL56, which indicates a novel molecular mechanism. Unexpectedly, this degradation was not observed in PRV infection assays using either wild-type (WT) or UL56-deleted (ΔUL56) PRV. Two BAG3 cleaved products were found in WT and ΔUL56 PRV infected cells in a species-specific manner. Overexpression and knockdown assays indicated that BAG3 played a negative regulation role during the PRV lytic infection in HEK293T cells.

## 2. Results

### 2.1. PRV pUL56 Interacted and Co-Localized with BAG3

To identify PRV proteins that interact with BAG3, we sought to analyze whether known protein−protein interaction modules were present in BAG3 and viral proteins. BAG3 is known as a WW domain-containing protein that interacts with the PPxY motif through a conformational binding [6,7,22]. Thus, the WW-domain−PPxY motif interaction module can represent a molecular basis for screening for viral proteins that interact with BAG3. Through amino acid (aa) sequence analyses, the pUL26 (^394^PPMY), pUL26.5 (^148^PPKY), pUL36 (^1534^PPKY), pUL41 (^345^PPNY), gD (^30^PPAY), and pUL56 (^74^PPSY, ^101^PPSY, ^112^PPAY, ^122^PPAY) were identified to be PPxY motif-containing PRV proteins. Among these proteins, pUL56 has four PPxY motifs, but only one for the others. Thus, pUL56 is a potential BAG3 interactor. 

**Figure 1:**
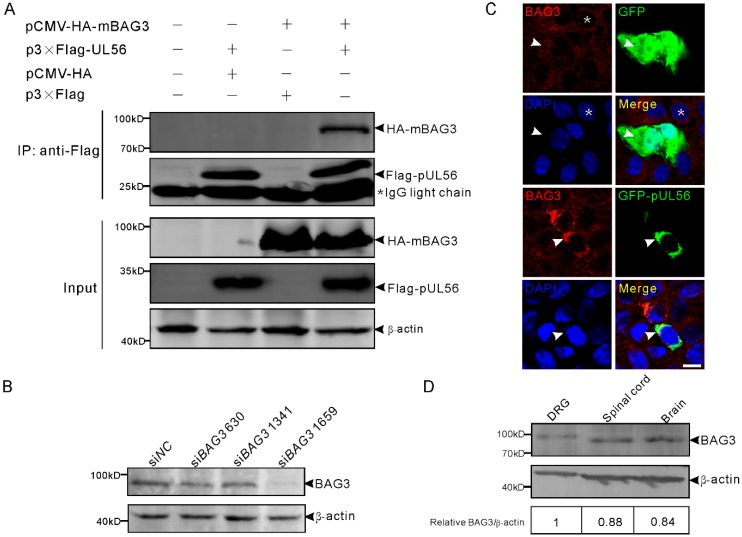
Pseudorabies virus (PRV) pUL56 interacts and co-localizes with BAG3. (**A**) Co-IP of pUL56 and BAG3. Each of the indicated plasmids is transfected into HEK293T cells, respectively. At 48 hpt, the whole cell lysates are obtained and mixed with the indicated combinations and immunoprecipitated with FLAG M2 beads. A 20% aliquot of whole cell lysates is used to confirm protein expression. The asterisk indicates a IgG light chain in the co-IP assay. (**B**) HEK293T cells in a 12-well plate are transfected with 120 pmol of the indicated siRNAs targeting to *BAG*3 for 48 h. The specificity of the anti-BAG3 polyclonal antibody is confirmed by a Western blot. (**C**) A co-localization analysis of endogenous BAG3 with GFP-pUL56 in the co-transfected Vero cells. BAG3 distributes evenly in the cytoplasm (asterisk, upper panel) in non-transfected Vero cells. The distribution of BAG3 is not affected by GFP (arrowheads, upper panel), whereas it is intensively restricted and co-localized with GFP-pUL56 in the perinuclear region (arrowheads, lower panel). (**D**) BAG3 can be detected in both the peripheral (dorsal root ganglion, DRG) and central (spinal cord and brain) nervous system in C57/BL6 mice. The scale bar indicates 10 μm (**C**).

We first examined whether pUL56 could interact with BAG3. The plasmids expressing Flag-pUL56 and HA-mouse BAG3 (mBAG3) were separately transfected into human embryonic kidney 293T (HEK293T) cells. At 48 h post transfection (hpt), the whole cell lysates were collected and mixed with FLAG M2 beads at 4 °C for 6 h. The co-immunoprecipitation (co-IP) result showed that HA-mBAG3 was specifically immunoprecipitated with Flag-pUL56, which indicates an interaction between pUL56 and BAG3 (Figure 1A). We continued to examine the co-localization between pUL56 and endogenous (endo-) BAG3 using a specific anti-BAG3 polyclonal antibody (Figure 1B). An evenly cytoplasmic dot-like distribution of BAG3 was observed, which was consistent with previous observations regarding the BAG3 distribution pattern in both cell cultures and tissues (Figure 1C) [23,24,25]. The representative confocal micrographs showed that endo-BAG3 intensively co-localized with GFP-pUL56, but not with green fluorescent protein (GFP) in the perinuclear region of transfected Vero cells (Figure 1C). Notably, the co-localization was nearly 100% observed in the GFP-pUL56 expressed cells.

Furthermore, the expression of BAG3 was detected in the mouse nervous tissues dorsal root ganglia (DRGs), spinal cord, and brain, which are the target organs infected by PRV (Figure 1D). This suggested a possible interplay between BAG3 and PRV in the nervous system.

### 2.2. PRV pUL56 Interacted with BAG3 through the PPxY Motifs

**Figure 2:**
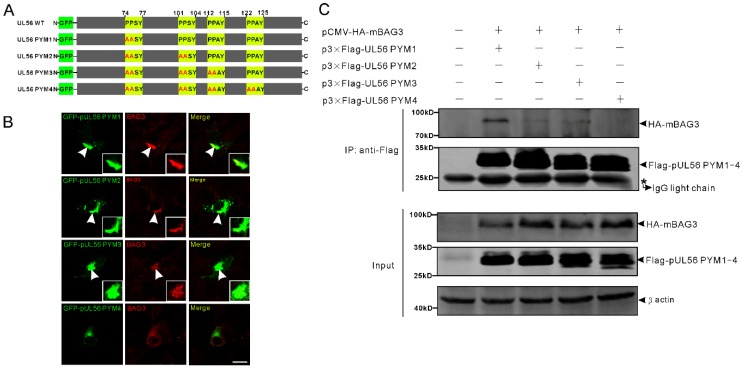
The pUL56 interacts with BAG3 through the PPxY motifs. (**A**) The schematic diagram shows four pUL56 PPxY mutants. (**B**) The co-localization analysis shows that pUL56 PYM1−3 co-localize with BAG3 in the transfected Vero cells, respectively. The pUL56 PYM4 has no apparent co-localization with BAG3 in the transfected Vero cells. The inset enlargements show the co-localized fluorescence in the cells. The arrowheads indicate co-localization. (**C**) Co-IP of BAG3 and pUL56 PYM1−4, respectively. A 20% aliquot of the whole cell lysates is used to confirm protein expression. The scale bar indicates 20 μm (**B**).

Next, we tested the hypothesis that pUL56 interacted with BAG3 through PPxY motifs. To achieve this goal, four pUL56 PPxY motif mutants PYM1 (^74^AASY), PYM2 (^74^AASY-^101^AASY), PYM3 (^74^AASY-^101^AASY-^112^AAAY), and PYM4 (^74^AASY-^101^AASY-^112^AAAY-^122^AAAY) were constructed as shown in Figure 2A. An intensive co-localization of BAG3 and GFP-pUL56 PYM1−3, but not GFP-pUL56 PYM4, was observed in co-transfected Vero cells (Figure 2B). Subsequently, the interactions between BAG3 and pUL56 PYM1−4 were analyzed by a co-IP assay. The result showed that the deletion of four PPxY motifs abrogated the interaction of pUL56 and BAG3 (Figure 2C).

### 2.3. PRV pUL56 Re-Localized BAG3 to Golgi

**Figure 3:**
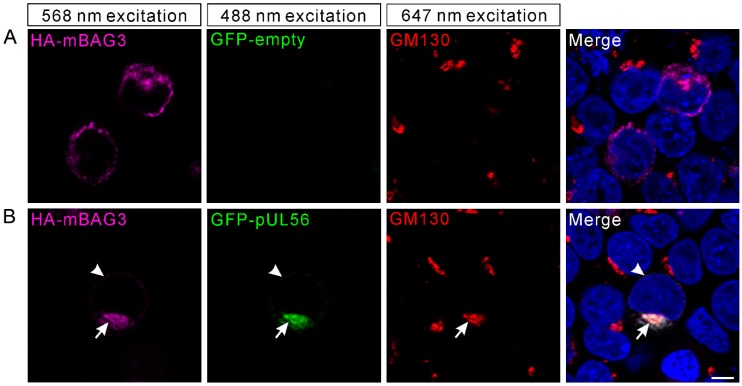
BAG3 is relocated to the Golgi via an interaction with pUL56. The plasmids pCMV-HA-mBAG3 and pAcGFP-UL56 are co-transfected into HEK293T cells. At 24 hpt, the HA-mBAG3 and Golgi are detected with anti-HA and anti-GM130 antibodies, which is followed by staining with Alexa Fluor 568-conjugate goat anti-mouse and Alexa Fluor 647-conjugate goat anti-rabbit secondary antibodies. (**A**) The HA-mBAG3 shows a cytoplasmic distribution in HEK293T cells. (**B**) A representative micrograph shows an intensive co-localization of HA-mBAG3 and GFP-pUL56, and both are targeted to the Golgi (arrows). In addition, partial GFP-pUL56 also targets HA-mBAG3 to the nuclear membrane (arrowheads). Nuclei are stained with 4′,6-Diamidino-2-phenylindole (DAPI). The scale bar indicates 5 μm (**A**,**B**).

We recently indicated that PRV pUL56 primarily localized to the Golgi complex and *tans*-Golgi network (TGN) [13]. Thus, we attempted to examine whether the interaction of pUL56 and BAG3 could specifically target BAG3 to the Golgi-TGN. HEK293T cells were co-transfected with plasmids pCMV-HA-mBAG3 and pAcGFP-pUL56 or pAcGFP. At 24 hpt, the transfected cells were fixed and stained with an anti-GM130 antibody. In the pCMV-HA-mBAG3 transfected cells, HA-mBAG3 was observed to have a cytoplasmic distribution without specific organelle localization (Figure 3A). However, HA-mBAG3 intensively localized to the Golgi upon co-expression with GFP-pUL56. In addition, some HA-mBAG3 was also anchored to the nuclear membrane by GFP-pUL56 (Figure 3B). Notably, the co-localization of HA-mBAG3, GFP-pUL56, and GM130 was nearly 100% observed in the co-expressed cells.

### 2.4. BAG3 Was Downregulated by the pUL56 C-Terminus

**Figure 4:**
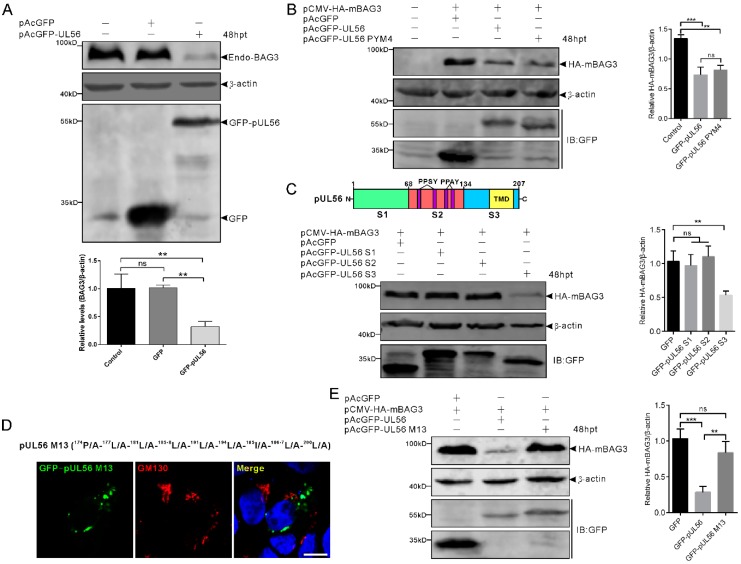
BAG3 downregulation is induced by the pUL56 C-terminus. (**A**) HEK293T cells are transfected with pAcGFP or pAcGFP-UL56, respectively. At 48 hpt, the endogenous BAG3 is significantly downregulated in pAcGFP-UL56, but not pAcGFP transfected or non-transfected cells. (**B**) Co-transfection of pCMV-HA-mBAG3 with pAcGFP, pAcGFP-UL56, or pAcGFP-UL56 PYM4 into HEK293T cells, respectively. At 48 hpt, HA-mBAG3 is significantly downregulated in pAcGFP-UL56 and pAcGFP-UL56 PYM4 as compared to pAcGFP transfected cells. In addition, there is no significant difference in the relative HA-mBAG3/β-actin levels between pAcGFP-UL56 and pAcGFP-UL56 PYM4 transfected cells. (**C**) The pUL56 is truncated into three segments S1 (1−67 aa), S2 (68−134 aa, containing four PPxY motifs), and S3 (135−207 aa, containing one predicted transmembrane domain, TMD). Co-transfection of pCMV-HA-mBAG3 with pAcGFP-UL56 S1, S2 and S3 into HEK293T cells, respectively. At 48 hpt, HA-mBAG3 is significantly downregulated in pAcGFP-UL56 S3, but not in S1 or S2 transfected cells as compared to the empty vector. (**D**) A pUL56 C-terminal mutant (M13) that loses Golgi localization in Vero cells. (**E**) Co-transfection of pCMV-HA-mBAG3 with pAcGFP-UL56 M13 does not affect relative HA-mBAG3/β-actin levels as compared to empty vector (right panel). The statistical analyses are performed using an ordinary one-way ANOVA post-Tukey’s multiple comparisons test (**: *p* < 0.01, ***: *p* < 0.001, ns: no statistical significance). Each statistical data represents three independent experiments. Scale bar indicates 10 μm (**D**).

To investigate whether the expression of BAG3 could be regulated by pUL56, 1 μg pAcGFP and pAcGFP-pUL56 was transfected into HEK293T cells, respectively. At 48 hpt, the protein levels of endo-BAG3 were determined by a Western blot. The relative level of endo-BAG3/β-actin was significantly downregulated in GFP-pUL56 as compared to GFP or non-transfected cells (Figure 4A).

Subsequently, we investigated whether the PPxY motifs played a role in pUL56-induced BAG3 downregulation. The pCMV-HA-mBAG3 was co-transfected with pAcGFP, pAcGFP-UL56, or pAcGFP-UL56 PYM4 (1 μg per plasmid) into HEK293T cells, respectively. At 48 hpt, the relative levels of HA-mBAG3/β-actin were analyzed. Both GFP-pUL56 and GFP-pUL56 PYM4 caused a significant downregulation of HA-mBAG3 as compared to GFP in co-transfected cells. This result indicated that the PPxY motifs and interaction of BAG3 and pUL56 did not contribute to pUL56-induced BAG3 downregulation (Figure 4B). Moreover, none of the PPxY motif-containing PRV proteins (pUL26, pUL26.5, pUL36 PY domain, pUL41, and gD) could modulate the levels of endo-BAG3, which further confirms that the PPxY motif was nonessential for modulating BAG3 expression (Supplementary Figure S1).

The pUL56 was subsequently truncated into three segments S1 (1−67 aa), S2 (68−134 aa), and S3 (135−207 aa) throughout a full length sequence (Figure 4C) [13]. The pCMV-HA-mBAG3 was co-transfected with pAcGFP, pAcGFP-UL56 S1, S2, or S3 (1 μg per plasmid) into HEK293T cells, respectively. At 48 hpt, the relative levels of HA-mBAG3/β-actin were determined in each group. Statistical analyses showed that HA-mBAG3 was significantly downregulated by GFP-pUL56 S3, but not by GFP-pUL56 S1 or S2 as compared to GFP in co-transfected cells. This result suggested that pUL56 mediated BAG3 downregulation through the C-terminus (Figure 4C).

Given an important role of the pUL56 C-terminus in Golgi−TGN targeting and retention [13], it was essential to clarify the relationship between specific Golgi localization and BAG3 degradation induced by pUL56. Subsequently, the pCMV-HA-mBAG3 was co-transfected with pAcGFP, pAcGFP-pUL56, or pAcGFP-pUL56 M13 (encoding a pUL56 C-terminal mutant that has lost Golgi localization and retention), respectively (Figure 4D) [13]. At 48 hpt, the relative levels of HA-mBAG3/β-actin were determined in each group. Strikingly, the mutant GFP-pUL56 M13 could not mediate BAG3 downregulation. This result suggested that the mutant amino acids or/and Golgi retention could be the key factors affecting pUL56-induced BAG3 downregulation (Figure 4E).

### 2.5. BAG3 Degradation Was Induced by pUL56 C-Terminal ^181^L-^185^L via the Lysosome Pathway in a Golgi Retention-Dependent Manner

**Figure 5:**
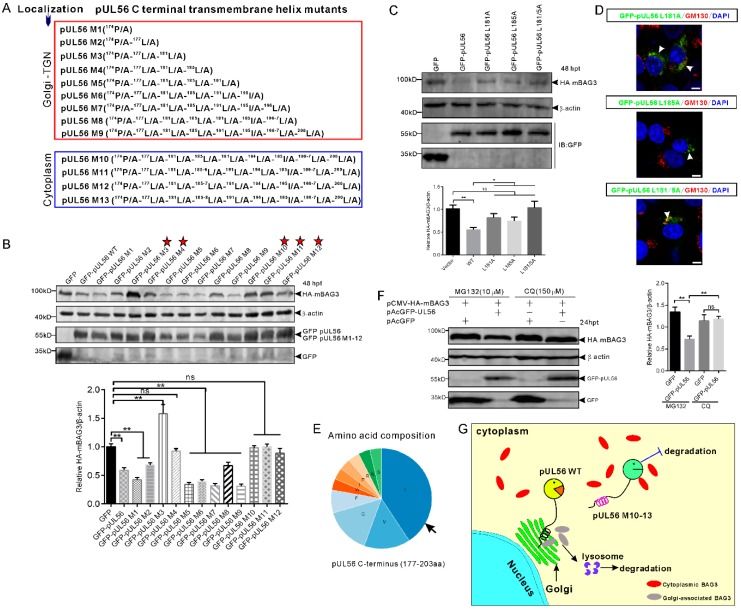
The C-terminal ^181^L-^185^L is responsible for pUL56-induced BAG3 degradation. (**A**) A series of pUL56 C-terminal mutants (pUL56 M1−13) were constructed in our previous study in which the M1−9 were shown to reside at the Golgi, whereas M10−13 resided in the cytoplasm. (**B**) HEK293T cells are co-transfected with 1 μg pCMV-HA-mBAG3 with an equal amount of pAcGFP, pAcGFP-UL56, or pAcGFP-UL56 M1−12, respectively. At 48 hpt, the whole cell lysates are collected and subjected to a Western blot analysis with the corresponding antibodies. The stars indicate the pUL56 mutants do not mediate HA-mBAG3 downregulation. (**C**) The mutations L181A, L185A, and L181/5A abrogate pUL56-induced BAG3 degradation. (**D**) The mutants pUL56 L181A, L185A, and L181/5A localize at the Golgi. The arrowheads indicate Golgi localization. (**E**) Analysis of amino acid composition of the predicted C-terminal transmembrane helix (177−203 aa) in pUL56. (**F**) The 1.5 μg pCMV-HA-mBAG3 is co-transfected with 1 μg pAcGFP or pAcGFP-UL56 into HEK293T cells, respectively. At 12 hpt, the cells are treated with the indicated inhibitors for 12 h. At 24 hpt, the cell lysates are collected and subjected to a Western blot analysis. (**G**) A schematic diagram depicts the molecular basis underlying pUL56-induced BAG3 degradation. Statistical analyses are performed using an ordinary one-way ANOVA post Tukey’s multiple comparisons test (*: *p* < 0.05, **: *p* < 0.01, ns: no statistical significance). Each statistical analysis represents three independent experiments. The scale bars indicate 5 μm (**D**).

The pUL56 C-terminal mutants (M1−12) were employed for further investigations of a precise molecular mechanism underlying the disrupted BAG3 degradation induced by pUL56 M13 (Figure 5A) [13]. Western blot results showed that HA-mBAG3 was significantly downregulated by WT, M1−2 and M5−9, but not by M3−4 or M10−12 (Figure 5B). Thus, these data indicated that the ^174^P/A-^177^L/A-^181^L/A-^185^L/A mutations were crucial in pUL56-induced BAG3 degradation. In addition, Golgi retention was also required because pUL56-induced BAG3 degradation was fully interrupted upon the loss of Golgi localization in pUL56 mutants M10−13. An unexpected finding was that HA-mBAG3 degradation was restored in pUL56 M5−9 as compared to M3 and M4 (Figure 5B).

Furthermore, the ^181^L and ^185^L were separately or together mutated into alanine, which resulted in pUL56 mutants L181A, L185A, and L181/5A. The pCMV-HA-mBAG3 was co-transfected with pAcGFP, pAcGFP-UL56, pAcGFP-UL56 L181A, pAcGFP-UL56 L185A, or pAcGFP-UL56 L181/5A (1 μg per plasmid) into HEK293T cells, respectively. At 48 hpt, the cell lysates were collected and subjected to a Western blot analysis. The result showed that HA-mBAG3 was significantly downregulated by GFP-pUL56, but not by GFP-pUL56 L181A, GFP-pUL56 L185A, or GFP-pUL56 L181/5A as compared to GFP in co-transfected cells (Figure 5C). Subcellular localization analyses indicated that GFP-pUL56 L181A, L185A, and L181/5A localized to the Golgi, similarly to GFP-pUL56 (Figure 5D). Based on these findings, we concluded that the leucine-rich pUL56 C-terminus contributed to pUL56-induced BAG3 degradation (Figure 5E).

To clarify the cellular degradation pathway involved in pUL56-induced BAG3 degradation, the lysosome and proteasome pathways were inhibited by MG132 and Chloroquine (CQ). Western blot results indicated that the lysosome pathway, but not the proteasome pathway, was responsible for pUL56-induced BAG3 degradation (Figure 5F). Taken together, we revealed a novel molecular mechanism in pUL56-induced host protein degradation, which occurred at the Golgi prior to proteins being transported into the cytoplasm (Figure 5G).

### 2.6. Endo-BAG3 Was Cleaved by PRV Infection in a Species-Specific Manner

**Figure 6:**
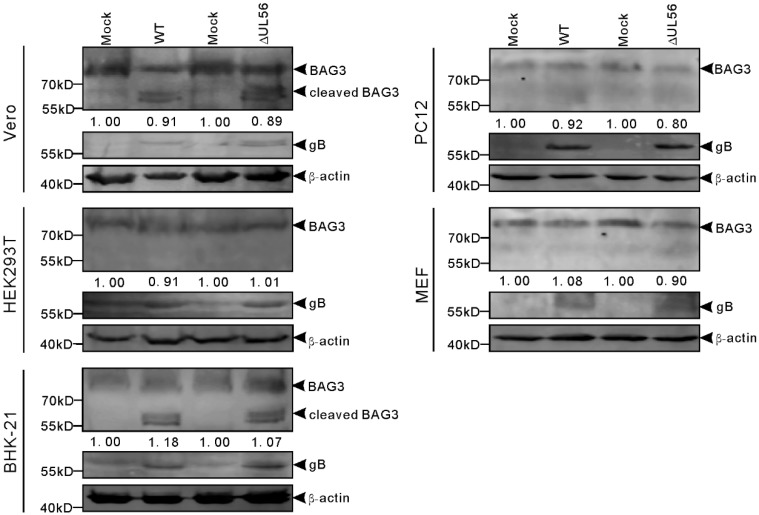
Cleaved BAG3 products are induced in pseudorabies virus (PRV) infected cells in a species dependent manner. The Vero, HEK293T, BHK-21, PC12, and MEF cells are infected with WT and ΔUL56 PRV at an MOI of 0.05, respectively. The mock infected cells are used as a control. At 24 hpi, the whole cell lysates are collected and subjected to a Western blot analysis with anti-BAG3 and anti-PRV gB antibodies.

Furthermore, we tested whether pUL56-induced BAG3 degradation also occurred in PRV infected cells. The cells were infected with WT or ΔUL56 PRV at the multiple of infection (MOI) of 0.05, respectively. At 24 h post infection (hpi), the relative endo-BAG3/β-actin levels were found to have no differences between mock and infection groups (Figure 6). Two adjacent BAG3 cleaved products with molecular weight around 60 kD were observed in PRV infected Vero and BHK21 cells. However, the BAG3 cleaved products were not found in HEK293T, PC12, or mouse embryonic fibroblast (MEF) cells (Figure 6). Moreover, this phenotype was not affected by pUL56 deficiency. Taken together, these data suggested that PRV-induced BAG3 cleavage had a species-dependent character, which was independent of pUL56.

### 2.7. BAG3 Played a Negative Regulation Role during PRV Lytic Infection

**Figure 7:**
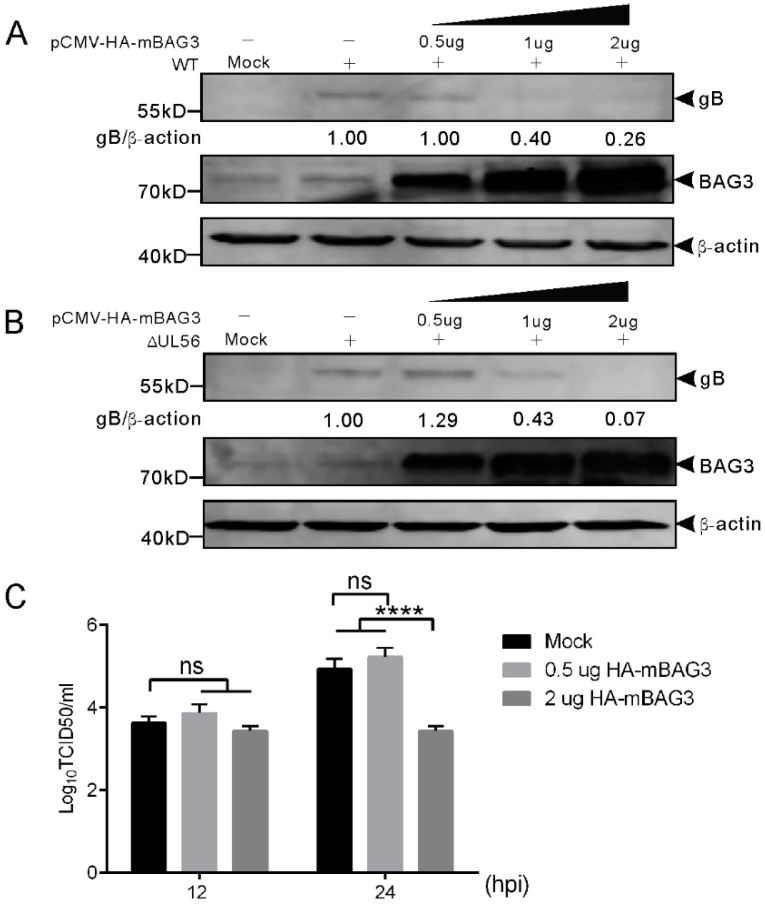
Overexpression of BAG3 suppresses PRV proliferation during lytic infection in HEK293T cells. (**A**,**B**) HEK293T cells are non-transfected or transfected with indicated amounts of pCMV-HA-mBAG3 for 24 h, which is followed by a mock-infection or infection with WT (**A**) or ΔUL56 (**B**) PRV at an MOI of 0.05. At 24 hpi, the whole cell lysates are collected and prepared for Western blot analyses. (**C**) HEK293T cells are non-transfected or transfected with 0.5 or 2 μg pCMV-HA-mBAG3 for 24 h, which is followed by infection with WT PRV at an MOI of 0.05. The intracellular viruses are harvested at 12 and 24 hpi, respectively. The viruses are titrated in Vero cells. The statistical analysis is performed using two-way ANOVA post Sidak’s multiple comparison test (****: *p* < 0.0001, ns: no statistical significance), and represents three independent experiments for titer determination.

To examine whether BAG3 overexpression could influence PRV proliferation, we transfected HEK293T cells with plasmid expressing HA-mBAG3 at different amounts (0.5, 1, and 2 μg). This was followed by infection with 0.05 MOI of WT and ΔUL56 PRV, respectively. At 24 hpi, the BAG3 and PRV gB proteins were detected by using a Western blot. The results showed overexpression of HA-mBAG3 at higher levels significantly suppressed the PRV gB protein level as compared to the non-transfection group in both WT and ΔUL56 infected cells (Figure 7A). The intracellular viral titers of WT were examined in HA-mBAG3 overexpressed and mock transfected groups at 12 and 24 hpi, respectively. The results indicated significantly lower viral titers were observed in 2 μg than 0.5 μg pCMV-HA-mBAG3 transfected or mock transfected cells at 24 hpi, which indicated a negative regulation role of BAG3 during PRV infection (Figure 7B).

**Figure 8:**
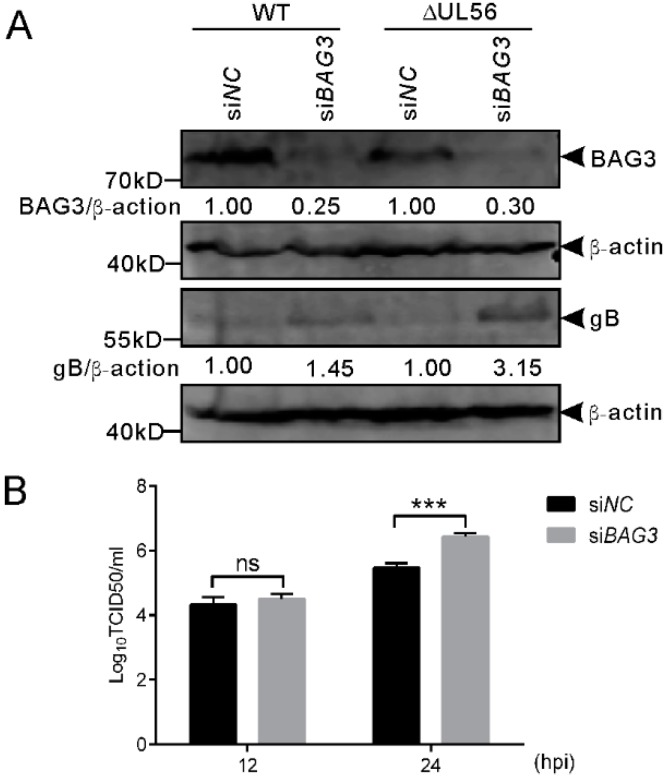
Knockdown of BAG3 promotes PRV proliferation during lytic infection in HEK293T cells. (**A**) HEK293T cells grown in a 12-well plate are transfected with si*BAG3* or si*NC* at a final amount of 120 pmol for 48 h, which was followed by infection with WT or ΔUL56 PRV at an MOI of 0.05, respectively. At 24 hpi, the whole cell lysates are collected and prepared for Western blot analyses. (**B**) HEK293T cells grown in a 12-well plate are transfected with si*BAG3* or si*NC* at a final amount of 120 pmol for 48 h, which is followed by infection with WT PRV and MOI of 0.05. The intracellular viral titers are determined by TCID_50_ in Vero cells at 12 and 24 hpi, respectively. The statistical analysis is performed using two-way ANOVA post Sidak’s multiple comparison test (***: *p* < 0.001, ns: no statistical significance) and it represents three independent experiments for titer determination.

To further confirm this finding, we performed an RNAi experiment to examine if the downregulation of BAG3 could promote PRV proliferation. The siRNA 1659 specific for BAG3 was transfected into HEK293T cells for 48 h, which is followed by infection with WT and ΔUL56 PRV at an MOI of 0.05, respectively. At 24 hpi, the BAG3 and PRV gB proteins were detected by a Western blot. As expected, the BAG3 levels were significantly lower in si*BAG3* than si*NC* transfected groups. The gB protein levels were significantly higher in si*BAG3* than si*NC* transfected groups (Figure 8A). The intracellular viral titers of WT were significantly increased in si*BAG3* than si*NC* transfected groups (Figure 8B). However, no significant differences were observed in viral titers at 12 hpi in either overexpression or knockdown assays (Figure 7 and Figure 8). Collectively, these results supported a negative role of BAG3 during PRV lytic infection. 

## 3. Discussion

Viruses have evolved various strategies to modulate and further utilize host protein machineries for their efficient replication and assembly. The modulations generally take place via the interplay between virus and host proteins, and, thus, become potential targets for designing antiviral tools.

*BAG3* is a stressor-response gene that has been reported to be modulated by a series of stress-inducers including high temperature, heavy metals, oxidative stress, and viral infection [12,26,27,28]. Host co-chaperone protein BAG3 can be regulated by a variety of viruses at the gene or post-transcriptional levels, which produces significant effects on the viral life cycle. For example, the expression of BAG3 can be inhibited by polyomavirus JC (JCV), which is a human neurotropic virus, as a result of suppressed transcription of the *BAG3* promoter through physical interaction with the T antigen in the infected glial cells or transfected human glioblastoma cells [29]. In turn, an overexpression of BAG3 suppresses the replication of JCV [30]. Conversely, the efficient replication in some viruses, e.g., VZV and SARS-CoV, relies on the presence of BAG3 [9,12]. During viral infection, the SARS-CoV significantly upregulates BAG3, but HSV1 does not substantially alter BAG3 within 12 h at the protein level [9,10]. Thus, these findings suggest that BAG3 can be either a positive or a negative regulator during viral infection.

Previous studies showed that the WW-domain bearing BAG3 is an interactor of the PPxY motif containing viral proteins, which provides a molecular basis for screening for BAG3 interacting viral proteins [7,11,31]. Based on this protein−protein interaction module, pUL56 was first identified to be a BAG3 interacting PRV protein in this study. 

Although significant amino acid differences are present among pUL56 homologs in alpha-herpesviruses (e.g., PRV vs. HSV1/2), they share many similar biological characteristics, such as Golgi-TGN localization, C-terminal transmembrane helix, and being a viral neurovirulence-associated factor [13,15,17,32]. In addition, the alpha-herpes viral pUL56 can induce a range of host protein (e.g., Nedd4, Itch, SLA1, and MHC-I) degradation through conserved PPxY motifs [16,18,19,20,33]. Differently, our findings indicated that pUL56-induced BAG3 degradation was independent of the PPxY motif (Figure 4B). Further evidence indicated that the C-terminus was responsible for pUL56-induced BAG3 degradation, which suggests a novel molecular mechanism. These results combined with our previous finding showing pUL56-induced non-interacted Rab6a degradation strongly supported that the interaction between pUL56 and its degraded protein was not required for pUL56 C-terminal-induced protein degradation [13]. Thus, it is impossible that the degraded BAG3 was transported as a cargo by interacting with pUL56 through vesicles to the degradation pathway. Given an important role of the pUL56 C-terminal leucine-rich transmembrane helix in Golgi retention [13], we, therefore, asked if pUL56-induced BAG3 degradation was in a specific Golgi localization and retention dependent manner. Using WT and sixteen pUL56 C-terminal mutants that retained or lost Golgi localization [13], we found that pUL56-induced BAG3 degradation was strongly correlated with the Golgi retention of pUL56. Of importance, the ^181^L-^185^L was responsible for the Golgi-resident pUL56-induced BAG3 degradation. In addition, the revertible BAG3 degradation induced by pUL56 M5-9 as compared to M3 and 4 remains to be clarified by using additional methodological approaches in the future work. Throughout this study, we used a series of pUL56 and its mutant overexpression assays to confirm the key residues contributing to the pUL56-induced BAG3 degradation. To our knowledge, there have been no papers showing an overexpression of alpha-herpes viral pUL56 could lead to cell apoptosis, cyctoxicity, or other unwarranted effects yet. Based on our data, we hypothesize that the abundant leucine at the C-terminus of pUL56 mutants could re-form the degradation-related sorting signal, which likely resembles ^181^L-^185^L in WT, via conformational contact. 

An unexpected observation was that BAG3 degradation could not be observed in the PRV infected cells. This suggests that potentially undefined mechanism does exist in antagonizing pUL56-induced BAG3 degradation during viral infection. However, two BAG3 cleaved products with similar molecular weight were observed in PRV infected cells, and this phenotype occurred in Vero (monkey) and BHK-21 (hamster) cells, but not in HEK293T (human), PC12 (rat), or MEF (mouse) cells, which suggests a species-specific manner. The cleavage of BAG3 was reported in a previous study, which showed a correlation with the apoptosis process in pancreatic cells [34]. The authors identified the C-terminal Asp 347 (KEVD347) as the cleaved site. The cleaved BAG3 has a molecular weight that seems to be much smaller than the ones observed in this study. Thus, the observed BAG3 cleaved products are likely derived from the N terminus in this study. In further work, the function of BAG3 cleaved products on PRV life cycle still needs to be clarified.

In the performed infection assays, HEK293T cells were employed to investigate the effects of BAG3 overexpression or knockdown on lytic infection of PRV. We used HEK293T cells because: (i) Human cell lines are susceptible to PRV, and support the efficient replication of PRV [35,36], (ii) HEK293T cells are highly efficient for overexpression of foreign protein by the transfected plasmid, and (iii) PRV has been reported in several recent case reports to infect humans, which results in encephalitis [37,38,39,40,41,42]. Both overexpression and knockdown assays indicated that BAG3 played a negative regulation role during PRV lytic infection. This conclusion was drawn by two findings: (i) Overexpression of BAG3 at an original transfection of 2 μg pCMV-HA-mBAG3 significantly decreased viral gB level and titer as compared to non-transfected cells at 24 hpi, (ii) knockdown of BAG3 significantly increased viral gB level and titer as compared to si*NC* transfected cells at 24 hpi. In an earlier study, the BAG3 was found to be a positive regulator during HSV1 infection through enhanced ICP0 activity [10]. This may also be true in PRV because the original transfection of 0.5 μg pCMV-HA-mBAG3 did not affect viral gB level (Figure 7A and B) and viral titer (Figure 7C) as compared to non-transfection cells. However, the suppression role of BAG3 could be observed in the original transfection of 1 or 2 μg pCMV-HA-mBAG3 as compared to the non-transfection group. It is also interesting to use an immediate early gene *IE180* deficient mutant to investigate if the positive regulation of BAG3 presents during PRV infection.

## 4. Materials and Methods

### 4.1. Cells and Viruses

HEK293T, PC12, BHK-21, and MEF cells were obtained from the American Type Culture Collection (ATCC). Vero cells were described in our previous study [43]. PC12 cells were differentiated in Dulbecco’s Modified Eagle’s Medium (DMEM; Thermo Fisher Scientific, Beijing, ChinaH supplemented with 1% horse serum and nerve growth factor (PEPROTECH, USA) at a concentration of 100 ng/mL. Differentiation medium was replaced every third day for 11 days before viral infection [44]. All cell lines were cultured in DMEM supplemented with 10% (*v/v*) heat-inactivated fetal bovine serum (Thermo Fisher Scientific, Beijing, China) and 1% penicillin-streptomycin, and cultured at 37 °C in a 5% CO^2^ environment. The WT and ΔUL56 PRV HeN1 strains (GenBank Accession No. KP098534) were propagated in Vero cells.

### 4.2. Plasmid Construction

The recombinant plasmids p3×Flag-UL56, pCMV-HA-UL56, pAcGFP-UL56, pAcGFP-UL56 S1, pAcGFP-UL56 S2, pAcGFP-UL56 S3, and pAcGFP-UL56 M1−13 were constructed in our previous study [13]. To construct recombinant plasmid pCMV-HA-mBAG3, the *BAG3* gene open reading frame was amplified from the reverse transcribed total mRNA of MEF cells using primers shown in Table 1, and then cloned into vector pCMV-HA. The PRV *UL26*, *UL26.5*, *UL41*, *gD,* and partial *UL36* (4150−5061 nt) genes were amplified from the HeN1 genome and cloned into vector p3×Flag using the primers shown in Table 1. The plasmid-expressing variant ^81^Met mBAG3 was used throughout this study. 

The UL56 mutants, PYM1−4, were amplified by overlapping extension PCR using mutant site-induced primers shown in Table 2, and subsequently cloned into vectors pAcGFP-C1 or p3×Flag, respectively. All constructs were validated by sequencing.

### 4.3. Generation of ΔUL56 PRV Using the CRISPR/Cas9 System

The use of the CRISPR/Cas9 system for the PRV double-stranded DNA genome editing was originally described in an earlier study [45]. To remove the *UL56* gene from the PRV genome, double sgRNAs targeting to 3′- and 5′-ends of the *UL56* gene were designed using an online CRISPR design tool (http://crispr.mit.edu/) (Supplementary Figure S2A). The plasmids expressing sgRNAs were constructed by inducing synthesized oligonucleotides into a *Bbs* I digested vector pX330 (Addgene) (Table 3). The recombinant plasmids pX330-sgRNA1 and 2 were validated by sequencing.

PRV gene editing induced by the CRISPR/Cas9 system requires the co-existence of the viral genome and CRISPR/Cas9 components [45]. Despite swine being the natural host of PRV, the human receptor nectin-1 recognized by PRV gD for cellular entry was found to have comparable efficiency with that of swine in PRV-mediated cell fusion [35]. Thus, the human cell line HEK293T was found to be susceptible for PRV, and was, therefore, used for pX330-sgRNA transfection to elevate the levels of intracellular sgRNAs. HEK293T cells grown to approximately 80% confluence in a 12-well plate were co-transfected with pX330-sgRNA1 and 2 (1 μg for each). At 24 hpt, the cells were infected with 225 plaque forming units (PFUs) of PRV. The supernatant was collected when approximately 50% of a cytopathic effect was observed. The obtained cell supernatant was serially diluted from 1 × 10^−1^ to 1 × 10^−4^. The 1 × 10^−4^ cell supernatant was inoculated into 100% confluent Vero cells in a 10-cm cell culture dish for viral plaque purification. The genome of the purified virus clone was extracted using an EasyPure Viral DNA/RNA Kit (TransGen Biotech, Beijing, China), according to the manufacturer’s instructions. Lastly, a pair of detection primers (UL56 KO F&R, Table 1) was used to verify the removal of the *UL56* gene by PCR (Figure S2B,C). The obtained ΔUL56 clone was subjected to three rounds of plaque purification to ensure that WT viruses were excluded. The deletion sites were identified by sequencing (Supplementary Figure S2D).

### 4.4. Antibodies

The following primary antibodies were obtained from commercial resources: mouse anti-Flag monoclonal antibody (F1804, Sigma-Aldrich), mouse anti-HA monoclonal antibody (H9658, Sigma-Aldrich), mouse anti-GFP monoclonal antibody (66002-1-lg, Proteintech), mouse anti-β actin monoclonal antibody (A2228, Sigma-Aldrich), rabbit anti-GM130 polyclonal antibody (11308-1-AP, Proteintech), and rabbit anti-BAG3 polyclonal antibody (10599-1-AP, Proteintech). The validations for the Golgi marker can be found in the website (GM130, http://www.ptgcn.com/Products/GOLGA2,GM130-Antibody-11308-1-AP.htm). The mouse anti-PRV gB monoclonal antibody (1E7) was prepared and stored in our laboratory. 

The following secondary antibodies were used for Western blot analyses: DyLight 800 labeled goat anti-rabbit IgG (H+L) and DyLight 800 labeled goat anti-mouse IgG (H+L) (KPL, MA, USA) for immunofluorescence analyses: Alexa Fluor 488 goat anti-mouse IgG (H+L), Alexa Fluor 568 goat anti-rabbit or mouse IgG; (H+L), and Alexa Fluor 647 goat anti-rabbit IgG (H+L) (Thermo Fisher Scientific, Beijing, China). The cellular nuclei were stained with 4′,6-Diamidino-2-phenylindole (DAPI; Thermo Fisher Scientific, Beijing, China).

### 4.5. Co-IP

HEK293T cells grown to approximately 80% confluence in six-well plates were transiently transfected with the indicated plasmids (2 μg for each), using the X-tremeGENE HP DNA Transfection Reagent (06366236001, Sigma-Aldrich), according to the manufacturer’s instructions. At 48 hpt, the transfected cells were harvested and lysed in RIPA lysis buffer (50 mM Tris, 150 mM NaCl, 1% NP40, 0.25% sodium deoxycholate, Beyotime Biotechnology, Shanghai, China) containing 1 mM PMSF and a complete protease inhibitor cocktail (Roche Diagnostics Gmb H, Mannheim, Germany) for 1 h on ice. Cell lysates were centrifuged at 12,000 × g for 10 min at 4 °C, and the obtained supernatants were used for immunoprecipitation with FLAG M2 beads (Sigma-Aldrich), according to the manufacturer’s instructions. Briefly, the supernatants were incubated with lysis buffer pre-washed FLAG M2 beads and incubated for 6 h under continuous shaking at 4 °C. The beads were rinsed three times with cold phosphate-buffered saline (PBS) (pH 7.4), and the immunoprecipitated proteins were separated by sodium dodecyl sulfate (SDS)-polyacrylamide gel electrophoresis (PAGE) followed by a Western blot analysis.

### 4.6. Viral Stocks and Titers 

Viral titers were determined by a PFU assay [46] or TCID_50_, according to the Reed-Muench method [47]. The PFU assay was performed as described in our previous study [46]. The HEK293T cells pretreated with the overexpression or knockdown of BAG3 were infected with 0.05 MOI WT and ΔUL56 PRV, respectively. At the indicated time points, the infected intracellular components were collected, and then subjected to one freeze-thaw cycle and centrifuged at 3000× *g* for 5 min to obtain the viral stock.

### 4.7. Animals and Tissue Preparation

Three six-week-old C57BL/6 mice were sacrificed, and the DRGs, spinal cord, and brain were dissected, and immediately put on dry ice. The samples were separately pooled and placed in lysis buffer (R0010, RIPA, Solarbio) containing 1 mM PMSF and a complete protease inhibitor cocktail, and were then properly sonicated on ice. The lysates were centrifuged at 12,000× *g* for 10 min at 4 °C. The supernatants were collected and the protein concentration was determined by a BCA protein assay kit (Thermo Fisher Scientific, Beijing, China). Laemmli samples containing approximate 20 μg protein were loaded in each lane and then subjected to a Western blot analysis. The animal experiment was approved by The Animal Ethics Committee of the Harbin Veterinary Research Institute (HVRI), Chinese Academy of Agricultural Sciences (CAAS) (Heilongjiang-SYXK-2006-032, 17 December 2017) approved the animal experiment. The animal experiment was performed in accordance with animal use ethical guidelines (American Psychological Association, APA).

### 4.8. RNAi

The siRNAs specific for human *BAG3* and the negative control were designed and purchased from Gene-Pharma (Shanghai, China). The siRNA sequences for human *BAG3* include BAG3-630 (sense, 5′-GCACCCUUUCCAUGUCUAUTT-3′; antisense, 5′-AUAGACAUGGAAAGGGUGCTT-3′), BAG3-1341 (sense, 5′-GGUGGAUUCUAAACCUGUUTT-3′, antisense, 5′-AACAGGUUUAGAAUCCACCTT-3′), BAG3-1659 (sense, 5′-CCUGAUGAUCGAAGAGUAUTT-3′, antisense, 5′-AUACUCUUCGAUCAUCAGGTT-3′), and a negative control siRNA (sense, 5′-UUCUCCGAACGUGUCACGUTT-3′, antisense, 5′-ACGUGACACGUUCGGAGAATT-3′). The HEK293T cells grown to approximately 40%−50% confluence in 12-well plates were transfected with the indicated siRNA at a final amount of 120 pmol using X-tremeGENE siRNA transfection reagent (Sigma-Aldrich) for 48 h, according to the manufacturer’s instructions.

### 4.9. Indirect Immunofluorescence Assay and Confocal Microscopy

Vero or HEK293T cells seeded in 20-mm glass-bottom cell culture dishes (NEST, China) were transiently transfected with the indicated plasmids. At 24 hpt, the transfected cells were fixed in 4% paraformaldehyde for 1 h at 4 °C. The cells were rinsed with PBS and the dishes were blocked in 3% bovine serum albumin (BSA) at 37 °C for 1 h. Subsequently, the cells were incubated with primary antibodies against BAG3 (1:500), HA (1:1000), or GM130 (1:1000) at 4 °C overnight. On the second day, the cells were rinsed three times with PBS, and incubated with Alexa Fluor 568-labeled and/or 647-labeled goat anti-rabbit or mouse IgG (H+L) diluted in PBS (1:1000) for 1 h at room temperature (RT). Lastly, the cells were counterstained with DAPI (1:4000) for 10 min at RT.

### 4.10. Western Blot

Protein lysates obtained from cells or tissues were separated on a 12% SDS-PAGE gel and transferred to a PVDF membrane. The membrane was blocked in 5% skim milk containing PBS-Tween 20 (PBST) for 2 h at RT, and followed by rinsing with PBST. Then, the membrane was incubated with primary antibodies diluted in PBS containing 5% BSA and 0.01% sodium azide at 4 °C overnight. Subsequently, the membrane was rinsed three times in PBST (20 min/time) and incubated with the corresponding secondary antibody (1:7500) for 1 h in the dark box under continuous shaking at RT. The membrane was rinsed three times with PBST (20 min/time). This was followed by detection with infrared imaging systems (Odyssey CLX).

### 4.11. Protein Degradation Pathway Inhibition Assays

The ubiquitin-proteasome and lysosome pathways are major systems for inducing protein degradation in cells. The inhibitors MG132 (Beyotime Biotechnology, Shanghai, China) and CQ (Sigma-Aldrich) were employed to inhibit the ubiquitin-proteasome and lysosome pathways, respectively. The HEK293T cells grown in a 12-well plate were transfected with 1.5 μg pCMV-HA-mBAG3 combined with 1 μg pAcGFP or pAcGFP-UL56. At 12 hpt, the transfected cells were treated with 10 μM MG132 or 150 μM CQ prior to harvesting. At 24 hpt, cell lysates were prepared as described in the co-IP assay and then subjected to a Western blot analysis.

### 4.12. Statistics

The statistical data were expressed as mean ± SEM. Statistical analyses were performed using GraphPad Prism 6.01 software (GraphPad Software, CA, USA). Quantification of the Western blot bands was performed by densitometry using ImageJ 1.48v software (NIH, MD, USA). The statistical analyses in Western blot were performed using an ordinary one-way ANOVA post-Tukey’s multiple comparison test. In the BAG3 overexpression and knockdown experiments, the difference of viral titers between treatment and control groups was analyzed by two-way ANOVA post Sidak’s multiple comparison test. Throughout the manuscript, *p* < 0.05 was used as the criteria for statistical significance (*: *p* < 0.05, **: *p* < 0.01, ***: *p* < 0.001, ****: *p* < 0.0001, ns: no statistical significance).

## 5. Conclusions

In this study, we identified PRV pUL56 as an interactor of host protein BAG3 through PPxY motif and WW domain interaction module. The pUL56 overexpression could result in a significant degradation of BAG3 through the lysosome pathway. Further site-specific mutagenesis assays mapped that the ^181^L and ^185^L contribute to pUL56-induced BAG3 degradation. This is different from previous reports showing alpha-herpesviral pUL56 homologs mediate host protein degradation through PPxY motif. Finally, a negative regulation role of BAG3 was found during PRV lytic cycle in both overexpression and knockdown assays. Collectively, our data provide a novel molecular mechanism on alpha-herpesviral pUL56-induced host protein degradation, and identify BAG3 as a host restricted factor during PRV lytic infection.

## Figures and Tables

**Table 1:** Primers used for recombinant plasmid construction or ΔUL56 detection in this study.

Primers	Sequence (5′-3′) ^*a*^	Constructs
mBAG3 HA F	GCGAATTCGGATGAGCGCCGCCACCCAATCGCC (*Eco* RI)	pCMV-HA-mBAG3
mBAG3 HA R	GCGGTACCCTAGGGAGCCACCAGGTTGCCAG (*Kpn* I)	pCMV-HA-mBAG3
UL26 Flag F	GCAAGCTTATGGGGCCCGTGTACGTGTCCGGC (*Hin* dIII)	p3×Flag-UL26
UL26 Flag R	GCGGATCCTCATCGGTGGGACATCATCTGGG (*Bam* HI)	p3×Flag-UL26
UL26.5 Flag F	GCAAGCTTATGGCAGCCCCGCCGAGCGCAGCG (*Hin* dIII)	p3×Flag-UL26.5
UL26.5 Flag R	GCGGATCCTCATCGGTGGGACATCATCTGGG (*Bam* HI)	p3×Flag-UL26.5
gD Flag F	GCAAGCTTATGCTGCTCGCAGCGCTATTGGC (*Hin* dIII)	p3×Flag-gD
gD Flag R	GCGGATCCCTACGGACCGGGCTGCGCTTTTAG (*Bam* HI)	p3×Flag-gD
UL41 Flag F	GCAAGCTTATGGGGCTCTTTGGCCTTTTAAAG (*Hin* dIII)	p3×Flag-UL41
UL41 Flag R	GCGGATCCTTATTTTCTCCTATGGGCGTTAC (*Bam* HI)	p3×Flag-UL41
UL36 PY Flag F	GCAAGCTTGAGTTCGCCGAGCGCATCGACGC (*Hin* dIII)	p3×Flag-UL36 PY domain
UL36 PY Flag R	GCGAATTCGCGGTGGCGCACGAAGCGCGCGAAGG (*Eco* RI)	p3×Flag-UL36 PY domain
UL56 KO F	GCGTACCCGATCGGTTGAGGTG	
UL56 KO R	GGGCGGGCGAGCCGAGTTTATTG	

*^a^* Underlined nucleotide sequence indicates restriction enzyme sites.

**Table 2:** Oligonucleotides used for mutagenesis of pUL56.

Primers	Sequence (5′-3′) *^a^*
UL56 PYM1	CGGCGCCCCCGGCTCCAGGGCGGCCTCCTACGGGGACGTCGTCCGCGTCG
UL56 PYM2	CGCTGTTTGCCCGGGGCCCGGCCGCGTCCTACTCGGAGACGCTCCTGTTCG
UL56 PYM3	GGAGACGCTCCTGTTCGACGCGGCCGCGTACGCGGTGACCATCCCGGACC
UL56 PYM4 UL56 L181A| UL56 L185A UL56 L181/5A	GCGGTGACCATCCCGGACGCGGCGGCGTACGAGCCCACCGTCATCGGGC CCGATGGTGCTCGTGGGCTTCGCCTGGGGGGGACTGCTCCTGCTGGTG CGTGGGCTTCCTCTGGGGGGGAGCGCTCCTGCTGGTGGGCCTCGTGTTTC CGTGGGCTTCGCCTGGGGGGGAGCGCTCCTGCTGGTGGGCCTCGTGTTTC

*^a^* Underlined nucleotide sequence indicates introduced mutant sites.

**Table 3:** Oligonucleotides used for sgRNA plasmid construction.

Oligos	Sequence (5′-3′)	Constructs
UL56 sgRNA1 F	CACCGGCCCCGCGGGCTCGTTGTGG	pX330-UL56 sgRNA1
UL56 sgRNA1 R	AAACCCACAACGAGCCCGCGGGGCC	
UL56 sgRNA2 F	CACCGACGACGGACTCCCGGAGCAC	pX330-UL56 sgRNA2
UL56 sgRNA2 R	AAACGTGCTCCGGGAGTCCGTCGTC	

## Supplementary Materials

Supplementary materials can be found at https://www.mdpi.com/1422-0067/21/9/3148/s1.
