# Supplementary material for: Host BAG3 Is Degraded by Pseudorabies Virus pUL56 C-Terminal 181L-185L and Plays a Negative Regulation Role during Viral Lytic Infection
Source: Int J Mol Sci. 2020 Apr 29;21(9):3148. doi: 10.3390/ijms21093148 (PMC7247713; doi:10.3390/ijms21093148)
Supplement: Supplementary file 1 [file ijms-21-03148-s001.zip › supplementary/supplementary/SI figures and legends 20200423.pdf]

Supplementary information

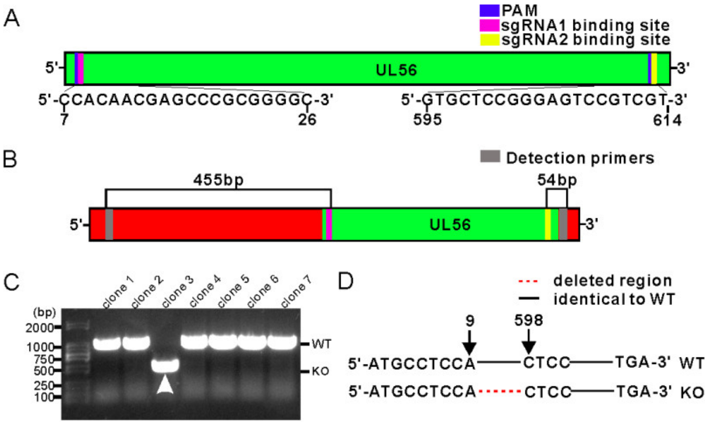

Figure S1. Construction and characterization of ΔUL56 PRV. (A) The PAM site-based sgRNAs are designed to target to 5'- and 3'-end of the *UL56* gene, respectively. (B) A pair of primers is used to detect the removel of *UL56* gene in PRV genome by PCR. (C) Clone 3 is a plaque-purified virus whose *UL56* gene has been successfully deleted. (D) DNA sequencing identifies the cleavage site (arrows) in the *UL56* gene of clone 3.

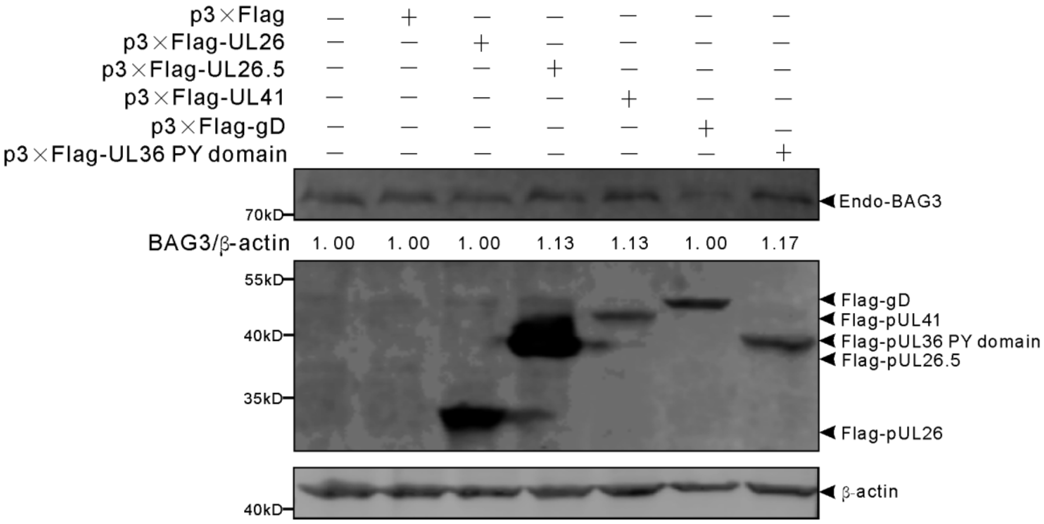

Figure 2. Expression of endogenous BAG3 cannot be modulated by the PPxY motif containing PRV proteins. The recombinant plasmids expressing PPxY motif containing PRV proteins pUL26, pUL26.5, pUL41, gD and pUL36 PY domain (1 μg per plasmid) are transfected into HEK293T cells, respectively. At 48 hpt, the endogenous BAG3 is detected with an anti-BAG3 polyclonal antibody, and the relative expression levels of BAG3/β-actin are analyzed using ImageJ.
