# Supplementary figures and images for: Host BAG3 Is Degraded by Pseudorabies Virus pUL56 C-Terminal 181L-185L and Plays a Negative Regulation Role during Viral Lytic Infection
Source: Int J Mol Sci. 2020 Apr 29;21(9):3148. doi: 10.3390/ijms21093148 (PMC7247713; doi:10.3390/ijms21093148)

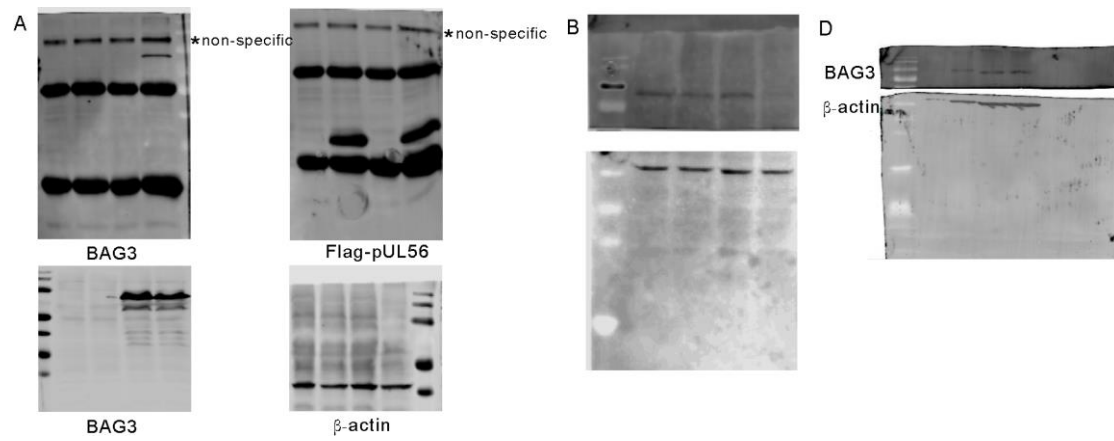

**Figure 1**

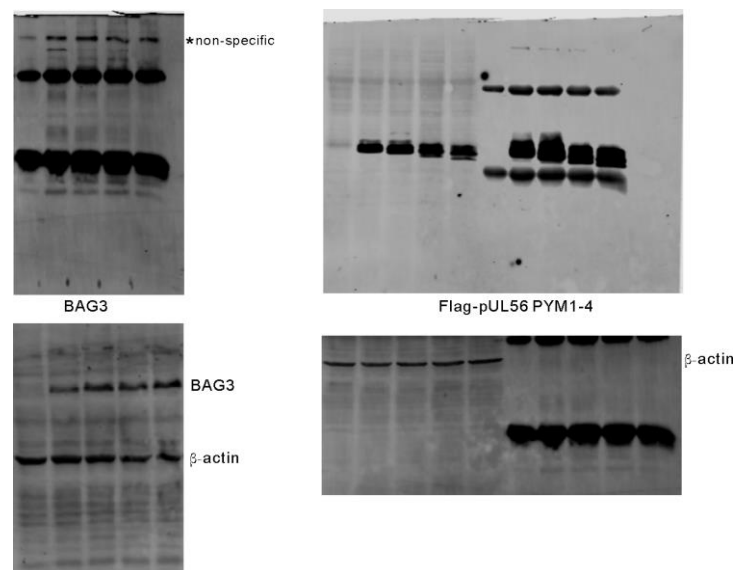

**Figure 2**

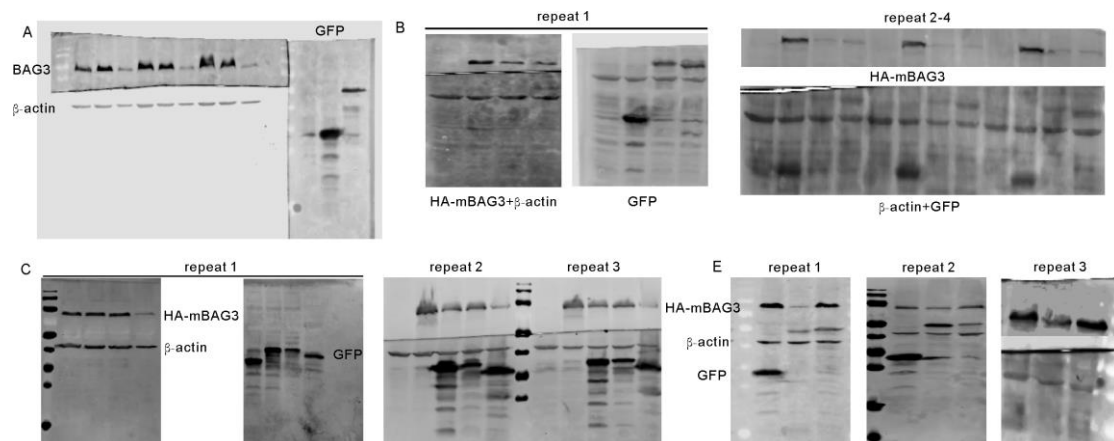

**Figure 4**

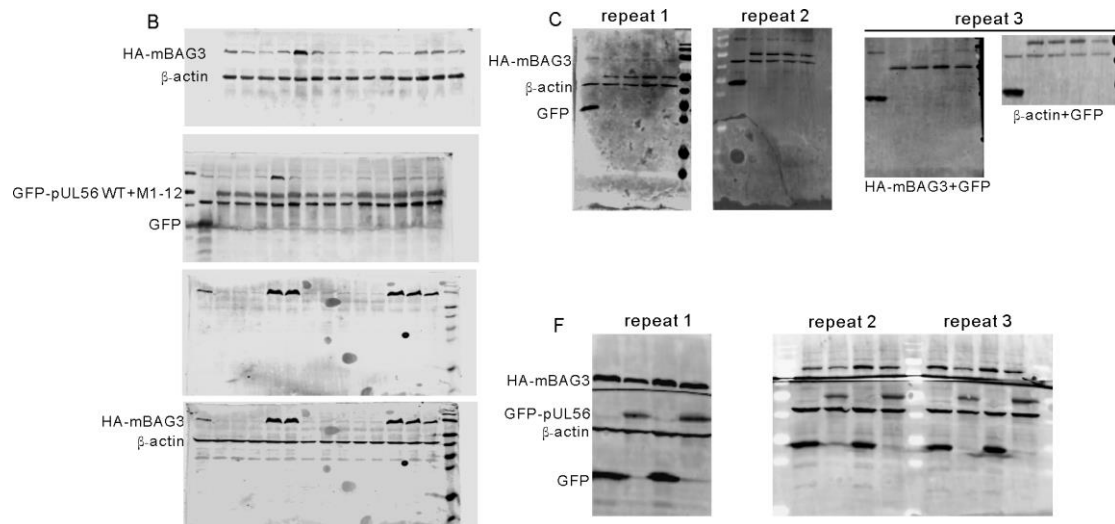

**Figure 5**

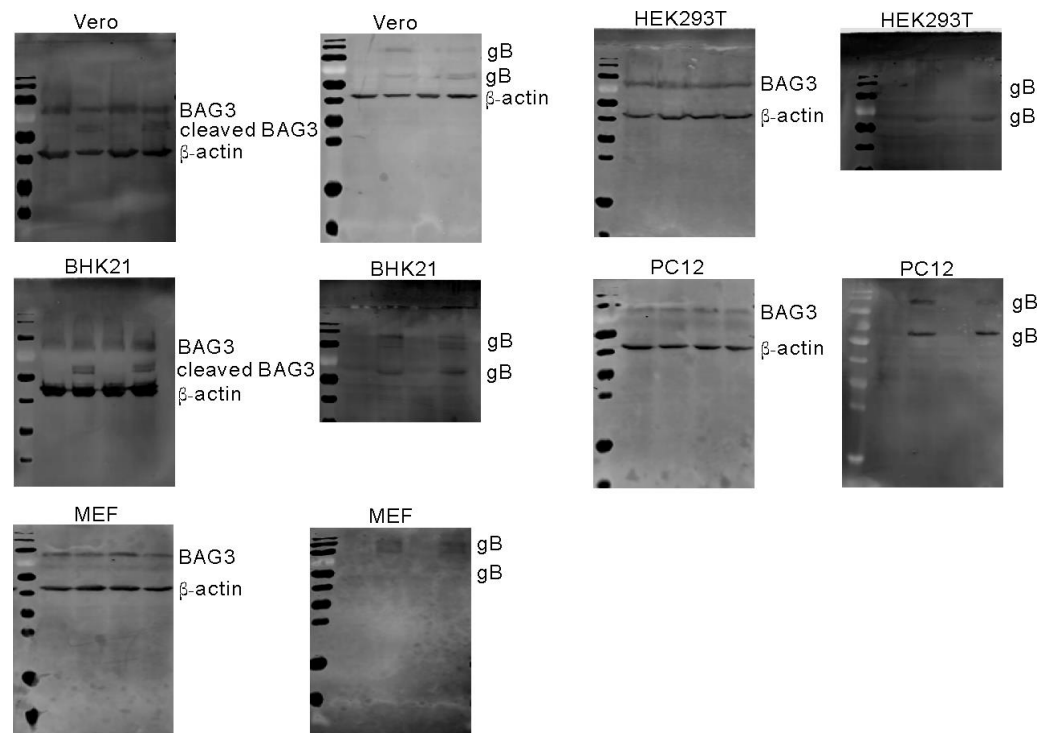

**Figure 6**

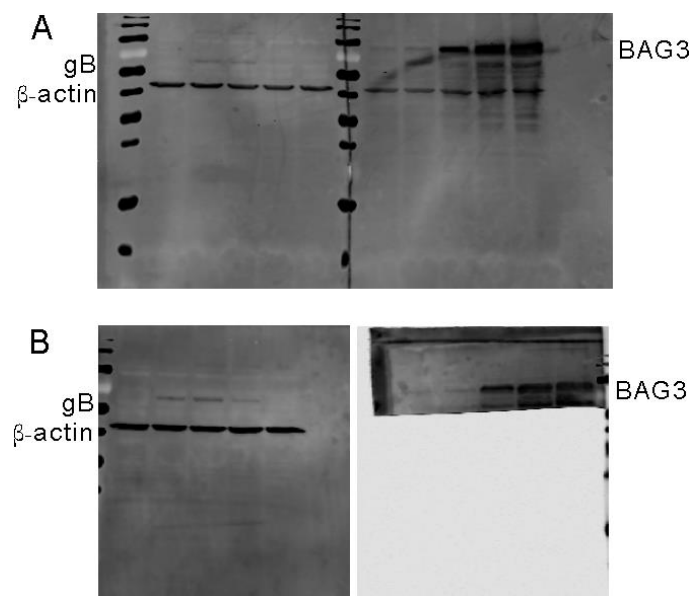

**Figure 7**

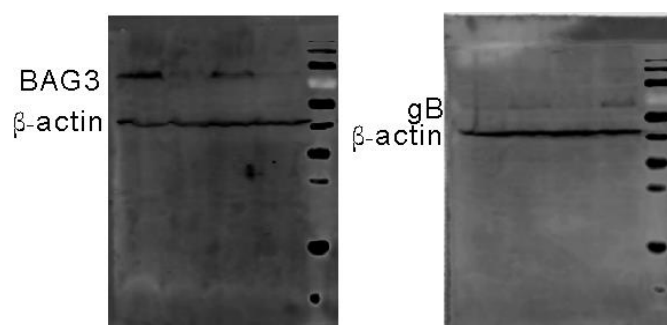

**Figure 8**

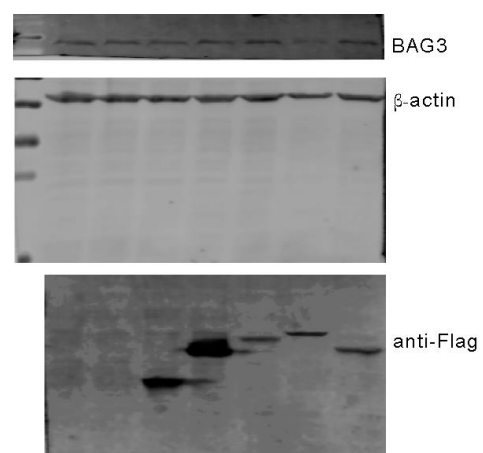

**Figure S1**

Supplement: Supplementary file 1 [file ijms-21-03148-s001.zip › supplementary/supplementary/Original images for WB.pdf]
